# Progress in Research on SARS-CoV-2 Infection Causing Neurological Diseases and Its Infection Mechanism

**DOI:** 10.3389/fneur.2020.592888

**Published:** 2021-01-13

**Authors:** Lintao Wang, Zhiguang Ren, Li Ma, Yanjie Han, Wenqiang Wei, Enshe Jiang, Xin-Ying Ji

**Affiliations:** ^1^School of Clinical Medicine, Henan University, Kaifeng, China; ^2^Henan International Joint Laboratory for Nuclear Protein Regulation and Kaifeng Key Laboratory of Infectious Diseases and Bio-safety, Henan University, Kaifeng, China; ^3^School of Basic Medical Sciences, Henan University, Kaifeng, China; ^4^Department of Infectious Diseases, Henan Provincial People's Hospital, Zhengzhou University People's Hospital, Zhengzhou, China; ^5^Clinical Laboratory, Functional Laboratory, Respiratory Department, Kaifeng Central Hospital, Kaifeng, China; ^6^Institute of Nursing and Health, School of Nursing and Health, Henan University, Kaifeng, China

**Keywords:** neurological complications, olfactorial nerve, ACE2, central nervous system, SARS-CoV-2, COVID-19

## Abstract

COVID-19 has spread rapidly worldwide since its outbreak and has now become a major public health problem. More and more evidence indicates that SARS-CoV-2 may not only affect the respiratory system but also cause great harm to the central nervous system. Therefore, it is extremely important to explore in-depth the impact of SARS-CoV-2 infection on the nervous system. In this paper, the possible mechanisms of SARS-CoV-2 invading the central nervous system during COVID-19, and the neurological complications caused by SARS-CoV-2 infection were reviewed.

## Introduction

The novel coronavirus is a previously unknown β-coronavirus, which is a single-stranded positive-strand RNA virus. The World Health Organization named it 2019-nCoV and the International Committee on Virus Taxonomy named it SARS-CoV-2. The virus belongs to a branch of the sarcoma virus subfamily of the coronavirus subfamily. SARS-CoV-2 is the seventh member of the coronavirus family that infects humans (1, 2).

SARS-CoV-2 is the virus that caused COVID-19 in 2019. SARS-CoV-2 infection can cause severe acute respiratory syndrome. SARS-CoV-2 has a high potential to spread and infect humans all over the world (3). Since the first case of COVID-19 was diagnosed in Wuhan, the number of SARS-CoV-2 infections worldwide has increased exponentially in the past few months. COVID-19 was originally described as a respiratory infection, but now it is increasingly regarded as a multi-organ disease, including nervous system manifestations. An updated version of the new guidelines for the diagnosis and treatment of coronary pneumonia issued by the National Health Council of China (China NHCotPsRo, 2020) points out that histopathological samples from some COVID-19 patients showed that SARS-CoV-2 invasion involved multiple organs, including lung, spleen and hilar lymph nodes, heart and blood vessels, liver and gallbladder, kidney, brain, adrenal gland, esophagus, stomach, and intestine. In particular, edema, and partial neuronal degeneration were observed in brain tissue (China NHCotPsRo, 2020) Neurodegenerative changes observed in cells infected with SARS-CoV-2, including cell death and hyperphosphorylation, as well as dislocation of Tau protein, these changes can be observed in conditions such as hyperthyroidism or Alzheimer's disease (4) However, the specific mechanism of neurodegenerative changes induced by SARS-CoV-2 remains to be further studied in the future. The central nervous system may serve as a reservoir for SARS-CoV-2, some groups observed that viral particles gradually accumulated within the neuronal cells of the brain organs from 6 to 72 h after SARS-CoV-2 infection, indicating that the virus replicated actively and effectively in the neuronal cells within the first few days of infection. However, some groups observed that viral infection did not replicate effectively in the first few days and suggested that the central nervous system might serve as a long-term reservoir of the virus (4). More and more evidence shows that SARS-CoV-2 has a potential neuroinvasive effect (5). It is estimated that more than 1/3 of COVID-19 patients will have nervous system symptoms, including central nervous system symptoms (dizziness, headache, disturbance of consciousness, acute cerebrovascular disease, ataxia, epilepsy). Peripheral nervous system symptoms (taste disorder, olfactory disorder, visual impairment, neuralgia) (6).

**Figure 1:**
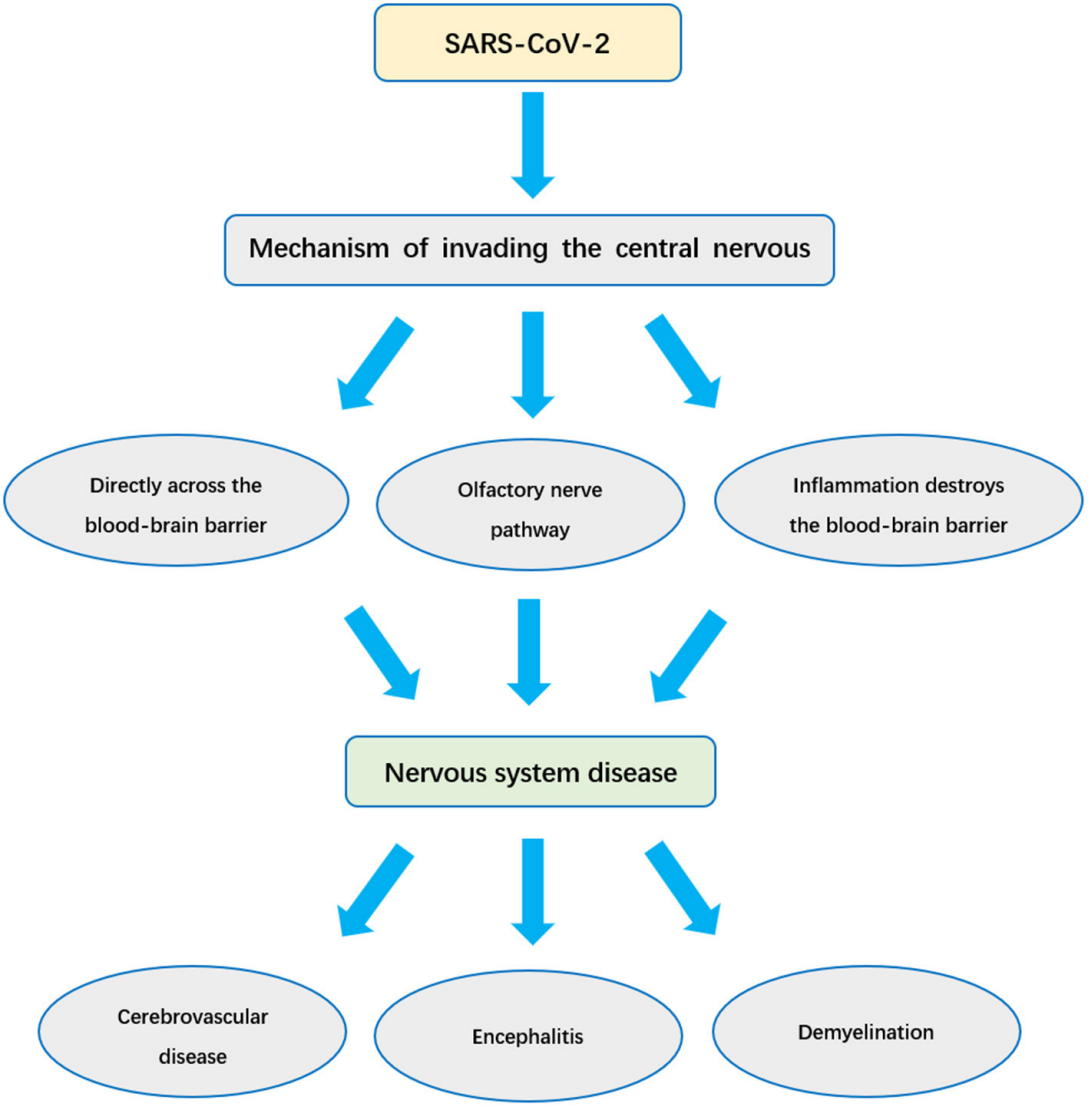
The mechanism of SARS-CoV-2 causing nervous system infection and nervous system diseases.

So far, although the epidemic in China has been effectively controlled, the COVID-19 epidemic is still very serious worldwide. According to statistics, there are more than 54 million confirmed cases worldwide, and more than 15 million existing confirmed cases. In this global public health emergency, we are still facing a very serious situation. In the face of SARS-CoV-2, understanding the impact of SARS-CoV-2 infection on the nervous system and its invasion mechanism is of great significance for the reasonable treatment of patients. In this paper, the effects of SARS-CoV-2 on nervous system are systematically analyzed and reviewed (Figure 1).

## Three Mechanisms of SARS-CoV-2 Invading the Nervous System

The central nervous system is protected by a highly complex brain barrier system, which is the first line of defense against virus invasion. The brain barrier is composed of the blood-brain barrier, blood-cerebrospinal fluid barrier, and brain-cerebrospinal fluid barrier. The blood-brain barrier has a maximum surface area that can be used for communication between the brain and blood. It consists of cerebral capillary endothelial cells, extracellular matrix, and astrocyte podocytes. The blood-cerebrospinal fluid barrier is located in the choroid plexus of the ventricle of the brain. The epithelial cells of the choroid plexus are mainly responsible for the barrier function of the blood-cerebrospinal fluid barrier. The blood-brain barrier and the blood-cerebrospinal fluid barrier can inhibit paracellular diffusion, protect the central nervous system from the influence of the constantly changing blood environment, infections and toxins, and are crucial for maintaining the homeostasis of the central nervous system (7–9).

To cause a central nervous system infection, the virus must first successfully cross the protective barrier of the brain. Crossing the blood-brain barrier or the blood-cerebrospinal fluid barrier requires special adaptation of the virus. Despite this, SARS-COV-2 can still enter the nervous system rapidly in some special ways after infection (10–13).

The following three ways may be the main ways for SARS-COV-2 to invade the central nervous system: (1) SARS-CoV-2 directly infects vascular endothelial cells by means of angiotensin converting enzyme 2, thus directly crosses the blood-brain barrier. (2) SARS-CoV-2 enters the central nervous system through synaptic connections via the olfactory nerve. (3) SARS-CoV-2 Induces Inflammation to destroy the Brain Barrier System and enter the central nervous system.

### SARS-CoV-2 Directly Infects Vascular Endothelial Cells and Crosses the Blood-Brain Barrier

Studies have shown that similar to SARS-COV, SARS-COV-2 can use ACE2 to enter the cell interior (14, 15). The spike protein (S protein) in SARS-CoV-2 is a trimer projecting from the viral membrane and contains a receptor binding domain (RBD) in each monomer. Through it, the viral protein can directly interact with ACE2 receptors on the surface of many host cells. S protein is the main tool for SARS-CoV-2 to bind to the ACE2 receptor (infect cells), and it can strongly bind to ACE2 (16). Therefore, the S protein may be used as a key target for the treatment of COVID-19 and vaccine development.

In addition, host cell protease also plays an important role in virus entry and infect cells (17, 18). The S1 subunit of S protein on the surface of SARS-CoV-2 first binds to neuron ACE2 receptor and adheres to the surface of target cells; then, the S2 subunit of the virus is activated by the serine protease TMPRSS2 of host cells, and the virus can enter the nerve cells (19). Therefore, TMPRSS2 activity is very important for the infection and transmission of SARS-CoV-2 in host cells and is important pathogenesis of neurological complications (20–22).

Although the S protein activity of SARS-CoV-2 is weaker than that of previous coronaviruses (23). However, the binding affinity of SARS-CoV-2 S protein to ACE2 is 10–20 times higher than that of SARS-CoV S protein to ACE2 (15). This is due to the fact that the receptor binding domain of SARS-CoV2 is different from that of previous coronaviruses on several key amino acid residues (17). It contains 4 positively charged residues and five negatively charged residues, so SARS-CoV-2 S protein is slightly more positively charged than SARS-CoV S protein. Although the charge difference between the S proteins of SARS-CoV-2 and SARS-CoV is quite small, this effect can be amplified by the presence of a large number of S proteins on the virus particles (16).

At the interface between the SARS-CoV-2 RBD and the cellular ACE2 receptor, the difference in the amino acid content of the S protein can lead to a more specific interaction between the S protein and the host cell receptor. Therefore, compared with SARS-CoV, SARS-CoV-2 is more likely to establish interactions with different targets in the human body through non-specific and specific interactions. All of these can ultimately increase the ability of SARS-CoV-2 to enter human cells (16), which may explain why COVID-19 has stronger pathogenicity, transmissibility, and greater global influence (17).

This charge difference between SARS-CoV-2 and SARS-CoV S proteins can have a significant impact on endothelial cell adhesion and crossing the blood-brain barrier (16) so that SARS-CoV-2 infected with vascular endothelial cells of the blood-brain barrier has a higher efficiency of reaching the brain and can cross the blood-brain barrier directly into the central nervous system (16).

Through the study of the distribution of ACE2 in the nervous system, it was found that ACE2 was expressed in different brain regions, such as subfornical organs, nucleus tractus solitarius and ventrolateral region of the medullary head, as well as regions such as motor cortex and raphe. According to spatial distribution analysis, ACE2 was also expressed in the substantia nigra (24–26). Due to the existence of ACE2 receptors in glial cells and neurons, it has become a potential target for SARS-CoV-2 causing brain injury and neurological symptoms (27).

In addition, ACE2 is widely attached to the extracellular surfaces of the lungs, arteries, heart, kidneys, intestines, and brain (cell membranes) (16, 28, 29). In addition to respiratory system involvement, SARS-COV-2 infection may also cause multi-organ dysfunction. Despite the predominance of respiratory symptoms, there is post-infection damage to the myocardium, kidneys, intestines, and liver, perhaps ACE2 provides a crucial link between immunity, inflammation, and cardiovascular disease (17).

The autopsy of novel coronavirus pneumonia showed brain edema and partial degeneration of neurons (30). However, there is not enough autopsy evidence to prove that SARS-CoV-2 exists in neurons and glial cells. Further studies are needed to prove this.

### Olfactory Nerve Pathway

**Figure 2:**
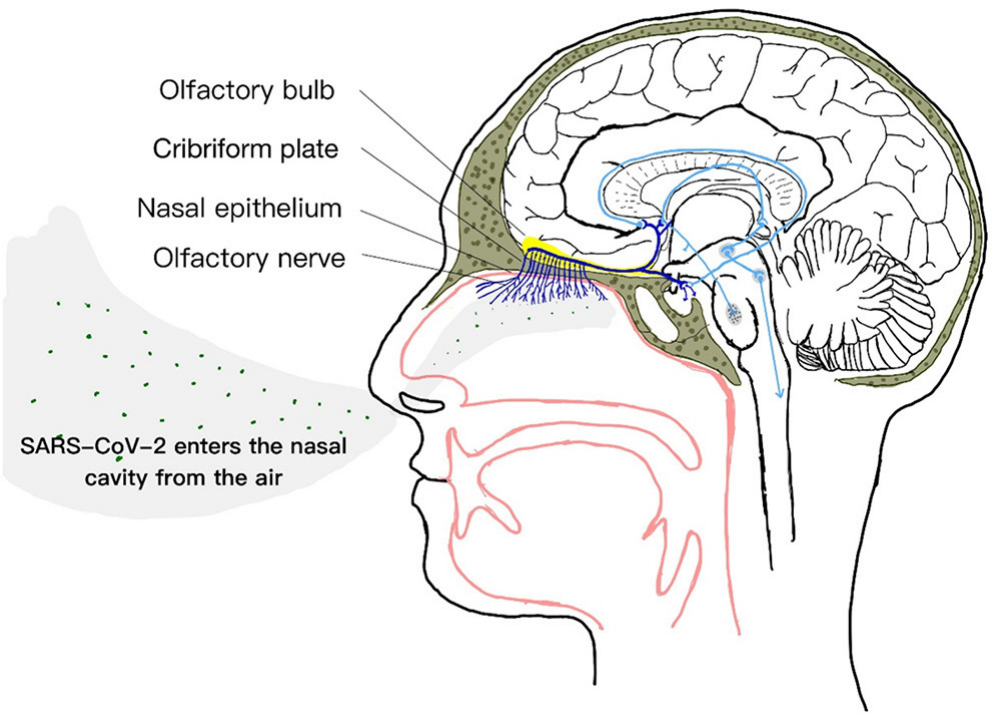
SARS-CoV-2 invades the brain through the olfactory nerve.

Among the 12 pairs of cranial nerves, the olfactory nerve is not a real nerve but a conduction bundle of the central nervous system. It can directly contact the brain (31), coupled with the special location and structure of the olfactory nerve itself, we speculate that perhaps in the mechanism of SARS-CoV-2 invading the central nervous system, the virus entering the central nervous system through the olfactory nerve is also one of the main ways (32, 33). And the olfactory nerve provides this way to enter the central nervous system, successfully bypassing the blood-brain barrier (34–36), effectively making it a channel between nasal epithelium and central nervous system. In the early stage of respiratory transmission, SARS-CoV-2 can enter the brain through the olfactory nerve (37) (Figure 2).

In the nasal cavity, the special olfactory neuroepithelium has an apical surface mainly composed of support cells, supporting the dendritic processes of neurons containing olfactory cilia (38). The dendrites of olfactory neurons are directly exposed in the airway of the nose (10). Although the olfactory system is very effective in controlling viral nerve invasion under normal conditions (39), however, data from several studies indicate that nasal respiratory epithelial cells express ACE2 and TMPRSS2 (31, 38, 40–42). As mentioned above, SARS-CoV-2 can enter the cell with the help of ACE2 and the serine protease TMPRSS2 of the host cell.

The expression of ACE2 and TMPRSS2 located in the olfactory neuroepithelium indicates a potential entry point for SARS-CoV-2 into the central nervous system (31), so that the virus can invade the olfactory nerve. The retrograde or anterograde transport of neurons is realized by kinesin and kinesin (37, 43, 44). During the infection process, SARS-CoV-2 uses the olfactory nerve to pass through the cribriform plate of the ethmoid bone to cause brain invasion (45, 46), which results in a rapid, transneuronal spread of the SARS-CoV-2 to relevant regions of the brain, which in turn interacts with ACE2 expressed on the surface of brain neurons (47–49). This cell tropism may be the reason why SARS-CoV-2 is highly infectious and related to olfactory dysfunction (38).

Because SARS-CoV-2 can directly act on nasal respiratory epithelial cells in the nasal cavity, olfactory dysfunction often occurs in the early stages of the disease. In mild to moderate cases, sudden loss of smell and taste is considered to be the strongest predictive symptom of early infection with the SARS-CoV-2 virus (50), and this mild, non-specific symptom can become asymptomatic. Or the only manifestation of a mildly infected person. The report of symptoms related to anosmia should be regarded as a sign of SARS-CoV-2 infection and a sign of COVID-19 (51, 52). If it is accompanied by transient brain edema and other neurological diseases, the first consideration should be the neuroinvasiveness of SARS-CoV-2 (38).

The olfactory dysfunction caused by SARS-CoV-2 may be explained by the following four mechanisms: ➀ Viral infections of the nasal mucosa can trigger inflammation of the nasal tissue, including the olfactory mucosa, thereby creating an obstructive barrier between odor chemicals and olfactory receptors; ➁ direct damage to olfactory receptors could prevent odor signals from being transmitted; ➂ the virus, being neurotropic, can attack the area of the brain responsible for smell along the path of the olfactory nerve; ➃ Loss of sense of smell may actually be a sequela of brain edema and partial neurodegeneration. Any or all of these four mechanisms may lead to loss of sense of smell in COVID-19 (53). Therefore, exploring the relationship between early loss of sense of smell and a long-term sense of smell has special clinical and prognostic value (54).

Although there have been many studies proving that the olfactory neuroepithelium expresses ACE2 and TMPRSS2, there are no sufficient and strong studies to prove that ACE2, TMPRSS2, and SARS-CoV-2 are widely present in the transmission bundle from the olfactory bulb to the CNS. In the future, further research is needed to resolve these inconsistencies, and more autopsy reports are needed to prove the presence of SARS-CoV-2 in the olfactory tract. Finally, the distribution of SARS-CoV-2 infected cells in human olfactory nerve conduction tracts was determined.

### Coronavirus Induces Inflammatory Responses to Disrupt the Blood-Brain Barrier System

The destruction of the blood-brain barrier through inflammation is also one of the ways for SARS-CoV-2 to enter the central nervous system. Infection with SARS-CoV-2 can destroy the blood-brain barrier by producing a large number of inflammatory mediators in the following three ways.

#### SARS-CoV-2 Directly Induces the Release of Cytokines by Immune Cells

When SARS-CoV-2 enters the human body, the virus activates immune cells, such as monocytes/macrophages, neutrophils, T cells, natural killer cells, and mast cells. The activated immune cells kill the virus by synthesizing and releasing cytokines (55–59). These cytokines mainly include interferon (IFN), interleukin (IL), chemokine, and tumor necrosis factor (TNF) (60). Some of their functions are to promote the inflammatory response and some to inhibit the inflammatory response. These cytokines are maintained in a balanced state in the healthy human body. Among them, pro-inflammatory factors can activate and recruit other immune cells, immune cells can secrete more cytokines, activate, and recruit more immune cells, thus forming a positive feedback cycle (55).

SARS-CoV-2 can cause immune cells to produce excessive immunity, cytokines are uncontrolled, a large number of cytokines are released, amplifying positive feedback, breaking the balance, marking an uncontrolled and dysfunctional immune response, leading to systemic inflammation, further aggravating the inflammatory response and increasing the severity of the disease. Although this excessive immune response can kill the virus, it can also cause some additional damage. Among them, the blood vessels suffered the most damage. Cytokine storms make the vessel wall more easily penetrated, and the blood-brain barrier is disrupted (61), causing neocoronavirus to enter the brain, inducing corresponding central nervous system symptoms.

#### Activation of Glial Cells Releases Proinflammatory Cytokines

Some neurotropic viruses can induce the pro-inflammatory state of glial cells and make them secrete cytokines (62). As mentioned earlier, glial cells express ACE2, We speculate that SARS-CoV-2, which enters the central nervous system through olfactory nerve, blood-derived, and other pathways, may also activate glial cells and induce a pro-inflammatory state (37, 63). In addition, experiments have confirmed that glial cells secrete a large number of inflammatory factors after being infected with coronavirus, such as interleukin-6, interleukin-12, interleukin-15, and tumor necrosis factor α, etc. (52). These cytokines can also damage the blood-brain barrier, further promote coronavirus to enter the brain, causing symptoms of central nervous system disease.

So far, the autopsy report on the glial cells of SARS-CoV-2 patients is still insufficient. We propose this possible mechanism based on the existing literature. In future studies, more autopsy reports are needed to prove whether SARS-CoV-2 infects glial cells through ACE2, or whether there are other receptors that can bind to SARS-CoV-2 in glial cells.

#### Vascular Endothelial Growth Factor Induces Inflammation

Vascular endothelial growth factor (VEGF) is widely distributed in the central nervous system (64). In addition, the combination of SARS-CoV-2 and ACE2 can activate the renin-angiotensin system which is involved in inflammation response, and then further promote the synthesis of VEGF through the binding of angiotensin II (AngII) and angiotensin II type 1 receptor (AT1R). In fact, in brain diseases, VEGF not only promotes angiogenesis but also destroys the blood-brain barrier by inducing inflammatory responses (64, 65).

**Figure 3:**
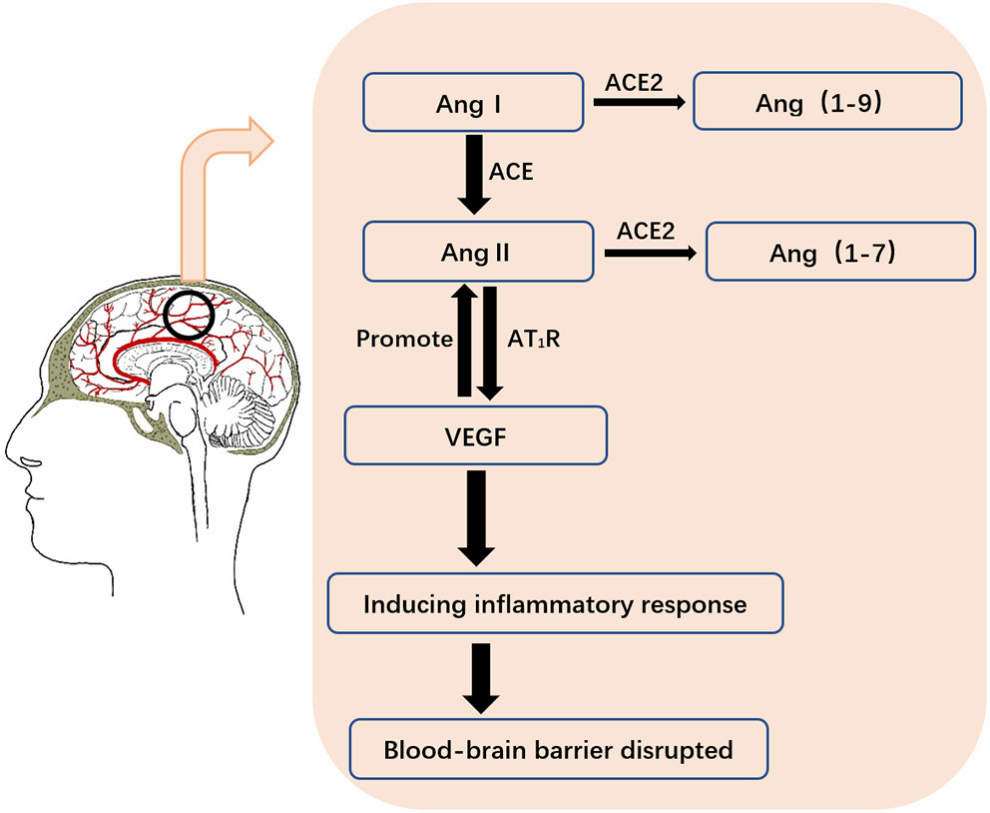
Inflammation induced by VEGF destroys the blood-brain barrier.

Inflammation is the precursor and companion of blood vessel formation, manifested by increased vascular permeability and recruitment of inflammatory cells (65). ACE2 is a key enzyme that catalyzes Ang I and Ang II to Ang 1-9 and Ang 1-7, respectively (66). When SARS-CoV-2 attacks ACE2, the inactivation of this enzyme can lead to the enhancement of the ACE/AngII/AT1R axis signal, followed by excessive AngII production. In the brain infected with SARS-CoV-2, the cumulative feedback of Ang II promoted the increase of ACE2. VEGF in turn reversely enhances Ang II, thus forming a vicious cycle in the release of pro-inflammatory cytokines, including TNF-α, IL-1β, IL-6, IL-8, and ICAM-1 (64, 67). In addition, among these cytokines, interleukin-6 (IL-6) is an important member of pro-inflammatory cytokines, which is positively correlated with the severity of COVID-19 symptoms. It may be used as one of the indicators of the severity of COVID-19 (37, 58) (Figure 3).

## Clinical Manifestations of Nervous System Diseases Caused by SARS-CoV-2

The neurological diseases possibly caused by SARS-CoV-2 can be divided into three major categories: ➀ Nervous system consequences of related lung and systemic diseases, such as cerebrovascular disease; ➁ The virus directly invades the central nervous system, such as encephalitis; ➂ Potential immune-mediated complications after infection, such as Guillain-Barre syndrome (GBS) and other types of demyelinating diseases.

Cerebrovascular disease refers to a group of diseases that occur in the blood vessels of the brain and cause brain tissue damage due to the disturbance of intracranial blood circulation.

Although the main manifestation of patients infected with SARS-CoV-2 is a lung disease, there are also cerebrovascular diseases (68). When the virus proliferates in lung tissue, it causes diffuse alveolar and interstitial inflammatory exudates, and even hyaline membrane formation. This will lead to abnormal alveolar gas exchange, hypoxia of the central nervous system, an increase of anaerobic metabolism of brain tissue, induction of intercellular edema, obstruction of cerebral blood flow, causing ischemia of cerebral circulation, with the increase of intracranial pressure, the brain function deteriorated gradually. It may even induce the occurrence of acute cerebrovascular disease, such as acute ischemic stroke (3, 37).

**Figure 4:**
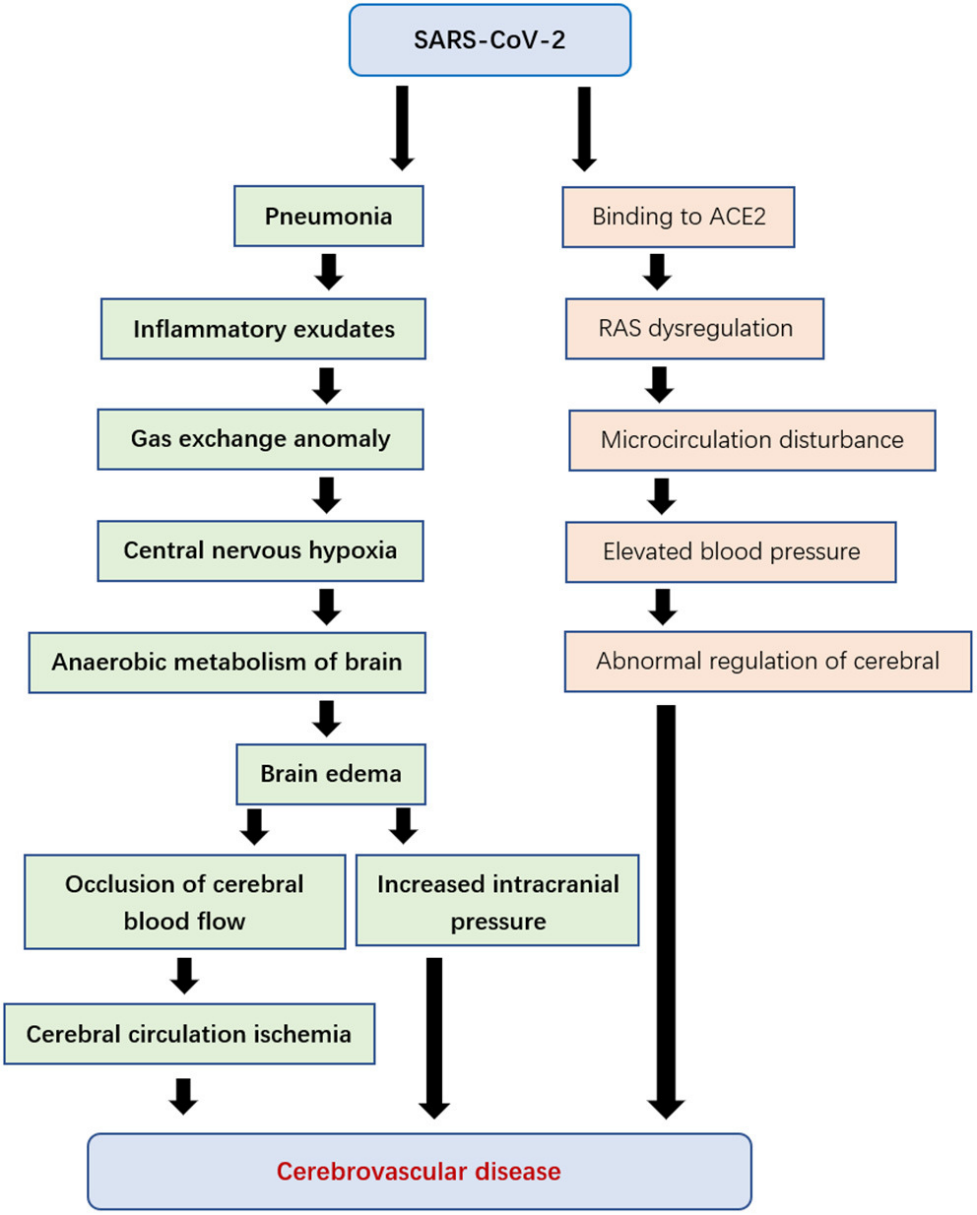
Cerebrovascular disease caused by SARS-CoV-2.

On the other hand, cerebral hemorrhage caused by elevated blood pressure may also be the result of the expression of ACE2 receptor (69, 70). ACE2 is one of the cardio-cerebrovascular protective factors, which plays an important role in regulating blood pressure and anti-atherosclerosis mechanism (71). SARS-CoV-2 may cause an imbalance of the renin-angiotensin system (RAS) by acting on the ACE2 receptor, leading to microcirculation disorder, affects cerebral blood flow regulation (72, 73), leading to an abnormal increase of blood pressure, and increases the risk of cerebral hemorrhage and ischemic stroke (Figure 4).

Encephalitis refers to the inflammatory lesions of brain parenchyma caused by pathogens, including neuronal damage and nerve tissue damage. It is characterized by acute episodes and common symptoms include headache, fever, nausea, vomiting, fatigue, convulsions, and disturbance of consciousness (37). At present, the presence of viral encephalitis in many patients infected with SARS-CoV-2 further speculated the existence of this neurological complication (74–77).

The treatment team of Beijing Ditan Hospital confirmed the presence of SARS-CoV-2 in the cerebrospinal fluid of patients infected with SARS-CoV-2 through genome sequencing, thereby clinically confirming viral encephalitis (37). This provides a solid foundation for SARS-CoV-2 to cause encephalitis. However, no signs of inflammation were found in brain tissue images of patients infected with SARS-CoV-2 (78). We guess that SARS-CoV-2 will produce virion vacuoles like MERS-CoV and SARS-CoV, if this hypothesis holds, then vacuolation may be a defense against infection. This problem needs further study and more pathological cases to clarify.

In patients with SARS-CoV-2, SARS-CoV-2 can stimulate immune cells to produce a variety of cytokines, resulting in an immune response process that causes nerve demyelination (79). For example, SARS-CoV-2 can cause Guillain-Barré syndrome (80, 81). Guillain-Barre syndrome, also known as acute idiopathic polyneuritis or symmetrical polyradiculitis, is an acute polyradiculoneuropathy (82). Clinical manifestations are progressive ascending symmetrical paralysis, quadriplegia, and varying degrees of sensory disorders (83). The exact pathogenesis of nerve demyelination caused by SARS-CoV-2 is not clear and remains to be further studied.

## Future Prospects

COVID-19 is a challenge to the world. At present, there is sufficient evidence that SARS-CoV-2 can invade the central nervous system and induce nervous system diseases. The possible pathways of SARS-CoV-2 invasion into the central nervous system include direct invasion of infected endothelial cells, invasion through the olfactory nerve, and invasion by inducing inflammation to destroy the brain barrier system. These pathways are all related to ACE2 receptors, so the relationship between SARS-CoV-2 and ACE2 should be further studied so as to take better measures to protect the central nervous system in patients with COVID-19. In fact, human respiratory viruses may also enter the central nervous system through other different ways, including the trigeminal nerve, cerebrospinal fluid, lymphatic system, and so on. The three mechanisms discussed in this article may be applicable to SARS-CoV-2, but we must be alert to other invasion mechanisms of SARS-CoV-2 until there is conclusive pathological evidence. In addition, it can be inferred from the existing data that encephalitis, cerebrovascular disease, nerve demyelination symptoms, and olfactory changes in COVID-19 patients are all likely to be related to SARS-CoV2 infection. These symptoms can be used as potential indicators of patient severity and prognosis. Understanding these knowledge is very important for the prevention and treatment of central nervous system symptoms and the rehabilitation of COVID-19 patients.

In addition, recent studies point out that other proteins expressed in nerve cells, such as Nrp1, may also become receptors for SARS-CoV-2. Future work needs to verify whether SARS-CoV-2 can invade the central nervous system using alternative receptors.

## Author Contributions

All authors listed have made a substantial, direct and intellectual contribution to the work, and approved it for publication.

## Conflict of Interest

The authors declare that the research was conducted in the absence of any commercial or financial relationships that could be construed as a potential conflict of interest.
